# Identifying Patients with Colorectal Cancer Likely to Benefit from a Trimodal Prehabilitation Prior to Surgery

**DOI:** 10.3390/nu18091369

**Published:** 2026-04-27

**Authors:** Nóra Suszták, András Fülöp, Lóránd László Lakatos, Dominic Herovi, Junghyun Cho, Petra Tímár, József Golub, Izabella Mihály, József Tamás Marton, Attila Szijártó, Balázs Bánky

**Affiliations:** 1 Faculty of Medicine, Semmelweis University, 1085 Budapest, Hungary; 2 Department of Surgery, St. Imre University Teaching Hospital, 1115 Budapest, Hungary; 3 Department of Surgery, Transplantation and Gastroenterology, Semmelweis University, 1085 Budapest, Hungary; 4 Department of Urology, University Hospital of Zürich, 8008 Zurich, Switzerland; 5 Department of Surgery, St. Borbala Hospital, 2800 Tatabánya, Hungary

**Keywords:** patient selection, trimodal prehabilitation, ERAS, enhanced recovery after surgery, colorectal surgery, colorectal cancer, trimodal prehabilitation, myosteatosis, enhanced recovery after surgery, ERAS, frailty, postoperative morbidity

## Abstract

**Introduction**: While enhanced recovery after surgery (ERAS) programs are widely implemented to reduce postoperative complications of colorectal cancer surgery, evidence for trimodal prehabilitation is inconsistent. We compared prehabilitation+ERAS versus ERAS alone, and explored patient subgroups most likely to benefit from targeted prehabilitation. **Methods**: A prospective, single-center parallel cohort study was conducted from October 2017 to August 2022. Consecutive adults undergoing elective colorectal surgery received ERAS alone or a 3–6-week trimodal prehabilitation programme (nutritional optimization, aerobic training, psychological preparation), followed by ERAS. Primary outcomes were overall postoperative morbidity at 7 and 30 days. Secondary outcomes included mortality, severe morbidity (Clavien–Dindo ≥ 3), 30-day readmission, and ICU/ward length of stay. **Results**: Of 344 screened patients, 244 were analyzed (ERAS n = 104; prehabilitation n = 140) with comparable baseline characteristics. Prehabilitation improved 6 min walk distance and incentive spirometry FVC by the time of surgery (*p* < 0.001 and *p* = 0.001, respectively), but between-group differences were not sustained at 8 weeks. Overall 7- and 30-day morbidity and mortality, severe morbidity, 30-day readmission, and length of stay did not differ between cohorts. In exploratory subgroup analyses, myosteatosis was associated with higher 7- and 30-day morbidity in the ERAS cohort (7% vs. 28% and 11.6% vs. 40%, respectively), whereas this contrast was not observed under prehabilitation. Among myosteatotic patients, prehabilitation was associated with lower 7-day morbidity (*p* = 0.045). Frailty was associated with severe morbidity, irrespective of allocation. **Discussion**: Trimodal prehabilitation improved preoperative functional measures but did not reduce short-term complications overall. CT-defined myosteatosis may help target prehabilitation to higher-risk patients.

## 1. Introduction

Colorectal cancer ranks as the third most prevalent type of cancer globally, accompanied by the second-highest mortality rate [1]. Although colorectal surgeries carry a considerable risk of postoperative morbidity and mortality, surgical resection stands as a cornerstone of intervention with curative intent. Despite a notable decline in fatality during the last five decades in developed nations, mortality associated with colorectal resection for the general population still remains relevant (8.6%) [2]. In addition, major morbidity remains at rates from 20 to 35% [3,4]. Besides the high rate of complications, functional impairment after a major abdominal surgery cannot be clinically neglected [5].

In an effort to mitigate postoperative morbidity in colorectal surgery, innovative surgical techniques such as laparoscopy and robotic surgery, along with enhanced recovery after surgery (ERAS) and trimodal prehabilitation programs, have been developed [6,7,8]. Trimodal prehabilitation programs are designed to optimize the nutritional, physical and mental health of patients prior to surgery [9]. These programs typically involve a combination of baseline assessment, counseling and optimization of nutritional, physical and mental well-being. While ERAS primarily focuses on reducing perioperative stress, prehabilitation aims to improve the stress tolerance of patients. However, it is essential to recognize stress tolerance differences among patients. Mitigating perioperative stress alone may not be sufficient for certain patient populations, as they may not possess the capacity to cope with it, potentially leading to detrimental outcomes. In addition, prehabilitation is a relatively expensive intervention requiring human resource capacity, compared to ERAS. Therefore, prehabilitation strategies should probably be tailored to specific patient groups on clinical terms.

While evidence supports the significant reduction in postoperative mortality and morbidity achieved through ERAS protocols in colorectal surgery compared to conventional treatment, the clinical benefit of prehabilitation programs is not straightforward for the entire, unselected colorectal patient population [7,10]. While a limited number of studies have demonstrated explicit reduction in mortality and morbidity across entire study populations, the majority of research has only been able to demonstrate the effectiveness of prehabilitation in improving functional parameters, such as the 6 min walking distance (6MWD) [11,12,13]. Our prior analysis found no significant improvement in postoperative complication rates [12], either.

To preserve the clinical relevance and cost-effectiveness of an established prehabilitation program, efforts were directed towards determining whether benefits are concentrated in specific subgroups of patients undergoing elective colorectal surgery, compared to an ERAS framework. While prehabilitation may improve functional measures at the population level, its routine use is only justified if it also translates into meaningful clinical endpoints, or if it can be targeted to those most likely to benefit. Accordingly, the aim of this study was to identify patient characteristics and pre-operative factors associated with differences in postoperative outcomes, particularly morbidity and mortality, in order to define clinically relevant subgroups for selective implementation.

## 2. Materials and Methods

### 2.1. Study Design

A prospective, single-center, parallel cohort study evaluating two perioperative care pathways differing in their nutritional and conditioning components was performed at Saint Borbala Hospital, a high-volume colorectal onco-surgery center in Tatabánya, Hungary, between October 2017 and August 2022. The study protocol complied with the ethical standards of the Declaration of Helsinki and received approval from the Hungarian Medical Research Council (ETT-TUKEB/IV/6746-3/2020/EKU). The trial was registered at ClinicalTrials.gov (NCT04595604). Written informed consent was obtained from every patient prior to study inclusion.

### 2.2. Patient Enrollment and Inclusion

**Figure 1:**
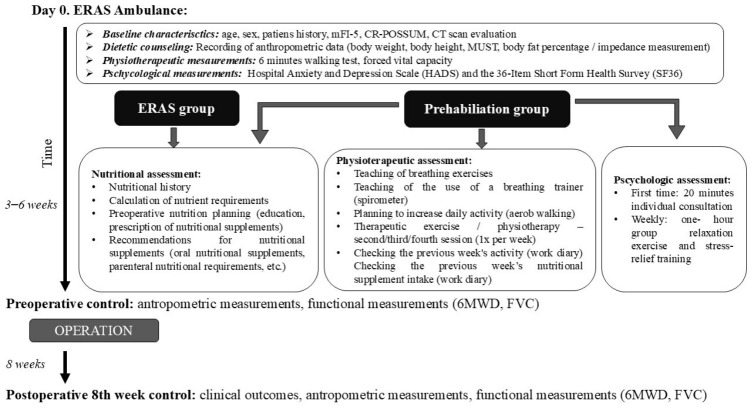
Overview of the study intervention.

All consecutive adult patients aged 18 years or older were eligible if they were scheduled for colorectal resection for malignant or benign disease or for colocolonic or colorectal reconstructive surgery. Patients were excluded if they underwent transanal minimally invasive surgery, emergency colorectal resections, palliative procedures or internal bypass due to peritoneal carcinomatosis, metastatic disease, irresectable tumors, or if they underwent small bowel reconstructive procedures. Eligible participants were included in one of two cohorts, as described previously by our group [12]. The ERAS cohort received baseline nutritional, physical and mental evaluation, ERAS counseling, two to four weeks of standardized oral nutritional supplementation in accordance with ESPEN recommendations, and standard perioperative ERAS management [14,15]. The prehabilitation cohort underwent the same baseline assessments and perioperative care, but additionally completed a three- to six-week trimodal prehabilitation program. The overview of the intervention is shown in Figure 1.

For both groups, a comprehensive dietetic assessment was performed on Day 0, during which all anthropometric and psychological data were collected. The assessment was conducted in person, in the presence of a dedicated ERAS nurse and a dietitian. Dietary habits were evaluated using a standardized questionnaire (see Supplement File S1). Anthropometric measurements, including height, body weight, body mass index (BMI), and body fat percentage, were subsequently obtained. Body fat percentage was assessed using a bioelectrical impedance body composition analyzer. Following the nutritional history assessment, the Malnutrition Universal Screening Tool (MUST) score was determined, and serum total protein and albumin levels were measured. Data were initially recorded in paper-based form, and subsequently entered in the REDCap electronic data capture system (version 14.0, Vanderbilt University, Nashville, TN, USA).

Based on these findings, individualized nutritional therapy recommendations were developed in accordance with previous ESPEN guidelines. Briefly, well-nourished patients with adequate oral intake did not receive specific oral nutritional supplementation, apart from the preoperative carbohydrate loading recommended by the ERAS protocol. Patients with insufficient oral intake but still able to drink received standard oral nutritional supplementation, targeting 25–30 kcal/kg/day for energy and 1.2–1.5 g/kg/day for protein concentration. Malnourished or severely malnourished patients received oral or enteral nutritional therapy. When neither oral nor enteral feeding was feasible, parenteral nutrition was recommended, with a target of 20–25 kcal/kg/day energy content with vitamin and trace element supplementation. From a dietetic and nutritional perspective, both cohorts received identical care. All patients underwent a baseline dietary consultation, during which they received a nutritional supplementation described above, together with education on healthy dietary practices (see Supplementary File S2).

As for the prehabilitation group, a physical assessment was performed, including the teaching of breathing exercises and the use of a breathing trainer (spirometer) and encouragement of daily moderate aerobic activity. Daily moderate aerobic activity is defined as physical activity performed at 3–6 METs, 65–75% of maximum heart rate, or a 5–6 on a 0–10 exertion scale. In practice, we advised participants to undertake a planned moderate-intensity brisk walking for at least 30 min daily, in addition to their routine everyday physical activity. Patient-reported moderate leisure activities, such as light cycling or gardening, were also enforced. The number of steps during the prescribed walk was monitored using a step-counting device.

Patients carried out these activities in a self-directed manner, supported by a booklet used to document daily walking, breathing exercises, incentive spirometer use, and nutritional supplement intake (please see Supplementary File S2). The completed booklets were reviewed on a weekly basis by the study physiotherapist. Patients were also encouraged weekly to maintain adherence to the prescribed regimen, and breathing techniques were reinforced during these assessments.

Psychological assessment included administration of the Hospital Anxiety and Depression Scale (HADS) and the 36-Item Short Form Health Survey (SF-36). During the first session, patients had an approximately 20 min individual consultation with a psychologist to identify potential psychological distress, including worries and anxiety. Also, support for the reduction in smoking and alcohol consumption was provided. Subsequently, patients attended a 1 hour long group session designed to practice stress management and relaxation techniques and to facilitate discussion of anxiety-related concerns.

For additional details, please refer to Supplementary File S2. Clinical staff delivering perioperative care did not direct pathway choice, and postoperative outcome assessors were blinded to cohort classification.

### 2.3. Assessment Schedule and Data Collection

At the initial preoperative outpatient visit, demographic and clinical characteristics, including age, sex, body mass index, body fat percentage, Malnutrition Universal Screening Tool (MUST) score, modified 5-item frailty index (mFI-5) and CR-POSSUM score were documented [16]. Briefly, CR-POSSUM (Colorectal Physiological and Operative Severity Score for the enUmeration of Mortality and Morbidity) is a colorectal surgery-specific risk assessment model used primarily to estimate perioperative, typically 30-day, mortality and to support the risk-adjusted audit of surgical outcomes. It incorporates six physiological and four operative variables, which are entered into a logistic regression equation to generate an estimated risk of death. We documented the CR-POSSUM score at pre-defined time points (baseline, on the day of operation) with the following online tool: https://www.evidencio.com/models/show/1775?v=1.9 (accesed on 1 January 2026) [17].

Functional performance was assessed using the six-minute walk distance (6MWD) and forced vital capacity (FVC). Psychological status and health-related quality of life were evaluated using the Hospital Anxiety and Depression Scale (HADS) and the 36-Item Short Form Health Survey. Preoperative computed tomography images were analyzed with OsiriX MD (version 14.0, Pixmeo SARL, Geneva, Switzerland) at the level of the third lumbar vertebra to determine the L3 skeletal muscle index, psoas muscle index and mean skeletal muscle Hounsfield units. Briefly, the psoas muscle index (PMI) is a computed tomography (CT)-derived surrogate marker of skeletal muscle mass, calculated by dividing the combined cross-sectional area of the right and left psoas muscles at the level of the third lumbar vertebra (L3) by height squared (cm^2^/m^2^) [18]. Hounsfield units (HUs) are relative measures of tissue radiodensity on computed tomography. For CT-based body composition analysis, we used the method described by Yoon et al. Skeletal muscle is segmented using a predefined attenuation threshold of −29 to +150 HU, and skeletal muscle index is calculated as skeletal muscle area divided by height squared (cm^2^/m^2^) [19]. Sarcopenia was defined as an L3 skeletal muscle index below 41 cm^2^/m^2^ in women or 53 cm^2^/m^2^ in men, while myosteatosis was defined as a mean muscle density below 27.5 Hounsfield units. The same anthropometric and functional evaluations were repeated on the day before surgery and at postoperative week eight, which served as the functional recovery time point. All data were stored in a secure REDCap database with automated pseudonymization. Surgeons, ward clinicians and outcome assessors remained blinded to cohort classification, and analyses were conducted on anonymized data.

### 2.4. Outcomes

The primary outcomes were seven-day and thirty-day overall postoperative morbidity. Secondary outcomes included seven-day and thirty-day all-cause postoperative mortality, seven-day and thirty-day severe morbidity (defined as Clavien-Dindo grade 3 or higher), thirty-day readmission rate, length of postoperative hospital ward stay and intensive care unit stay, and changes in functional capacity (expressed as differences in six-minute walk distance and forced vital capacity between baseline) at postoperative week eight. All outcomes were defined a priori and were ascertained uniformly in both cohorts.

### 2.5. Statistical Analysis

Continuous variables with normal distribution were analyzed with their mean ± standard deviation, while skewed distributed variables were reported as median with interquartile range (IQR) and minimum–maximum ranges. Categorical variables were reported as absolute numbers (n) with relative percentages (%) of the given group. Normal distribution of continuous variables was determined by the Shapiro–Wilk test. Continuous variables were compared using Student’s *t*-test or Mann–Whitney U-test, as appropriate. Categorical variables were compared using Chi-square test or Fisher’s exact test. To identify patient subgroups most likely to benefit from each intervention, associations between age, sex, body mass index, CR-POSSUM score, 6MWD, FVC, MUST score, mFI-5, sarcopenia and myosteatosis as dicho, and 30-day and 7-day overall mortality, overall morbidity and severe morbidity. Variables were dichotomized for these calculations. In addition, age, sex, intervention type, body mass index, CR-POSSUM score, 6MWD, FVC, MUST score, mFI-5, sarcopenia and myosteatosis were examined as independent predictors of the primary outcomes using univariable and multivariable logistic regression models. The maximum number of predictors was assessed by the 1–10 rule-of-thumb. Variables deemed the most clinically plausible or demonstrating an univariable association with the outcome at *p* < 0.10 were entered into a forward-stepwise multivariable logistic regression model. Only complete cases were used during modeling, and no post hoc variable selection or interaction terms were included. Continuous variables were tested for linearity-in-the-logit by the Box–Tidwell test. The model was fitted via the maximum-likelihood method. Unadjusted and adjusted odds ratios (ORs) with 95% confidence intervals (95% CIs) are reported for each predictor. Level of statistical significance was set at a two-tailed *p* value of <0.05. All statistical analyses were done using SPSS version 24.0 (SPSS Inc., Chicago, IL, USA). The study adheres to the STROBE Statement (STrengthening the Reporting of OBservational studies in Epidemiology, www.strobe-statement.org (accesed on 1 January 2026)).

## 3. Results

### 3.1. Patient Enrollment

**Figure 2:**
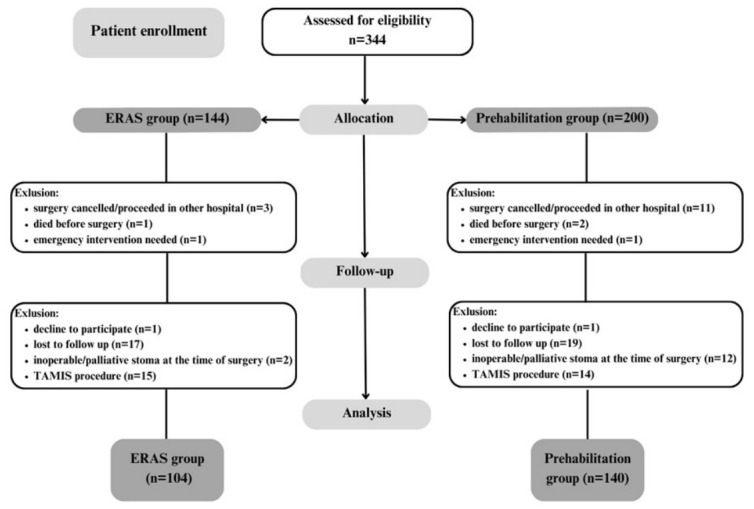
CONSORT (flow) diagram of study enrollment.

The patient enrollment process is summarized in the flow diagram (Figure 2). Over a 58-month study period, 344 patients were assessed for eligibility. Patients undergoing emergency or palliative surgery, those who died preoperatively, those whose planned procedure was canceled for any reason, and patients treated with transanal minimally invasive surgery (TAMIS) were excluded. Ultimately, 244 patients were included in the final analysis: 104 in the control group and 140 in the prehabilitation group.

### 3.2. Baseline Demographic and Clinical Characteristics

Baseline demographic and clinical characteristics are summarized in Table 1. Cohorts were broadly comparable with respect to age, comorbidity burden, CR-POSSUM score, BMI, body fat percentage, MUST score, mFI-5 score, sarcopenia status, L3 skeletal muscle and the psoas indices, and myosteatosis, with no statistically significant differences. Tumor pathological characteristics and operative procedures were likewise similar between cohorts. However, nodal staging differed, driven by a higher proportion of patients classified as pN0 in one group (Table 2).

### 3.3. Functional and Postoperative Clinical Outcomes

Relative to baseline, patients assigned to prehabilitation demonstrated a significantly greater improvement in 6MWD by the time of surgery than those receiving ERAS alone (121.0 ± 52.8% [range 0–376] vs. 106.0 ± 24.8% [range 63–206]; *p* < 0.001). This between-group difference was no longer evident at 8-week follow-up (101.7 ± 67.7% [range 0–368] vs. 107.4 ± 48.7% [range 0–233]; *p* = 0.876). A similar pattern was observed for incentive spirometry-derived FVC. At the time of surgery, the prehabilitation group achieved a significantly higher relative FVC than the control group (114.3 ± 39.4% [range 0–300] vs. 100.0 ± 18.8% [range 60–167]; *p* = 0.001), whereas no between-group difference was observed at 8 weeks. In contrast, objective spirometric FVC remained stable over the study period in both cohorts (Table 3).

No statistically significant between-group differences were observed in overall mortality or morbidity at either 7 or 30 days postoperatively. Postoperative intensive care unit length of stay, total hospital length of stay, and 30-day readmission rates were likewise comparable between the two study arms (Table 4). Multivariable logistic regression results are reported in Table 5. Overall model fit was not statistically significant for either the 7-day model (χ^2^ = 5.74, *p* = 0.57) or the 30-day model (χ^2^ = 7.88, *p* = 0.34). Although prehabilitation showed a protective association with overall postoperative morbidity at both time points (7-day: OR = 0.40, 95% CI 0.12–1.28, *p* = 0.12; 30-day: OR = 0.58, 95% CI 0.22–1.52, *p* = 0.27, it was not retained in the final models.

### 3.4. Subgroup Analysis

**Figure 3:**
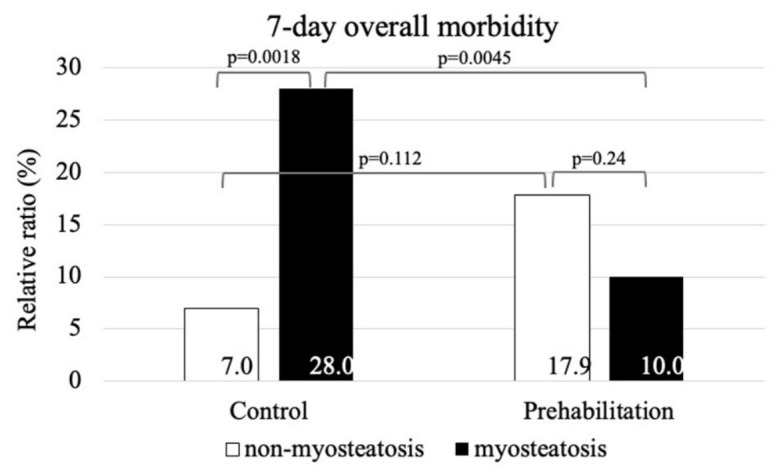
The 7-day overall morbidity. Cases are stratified by myosteatosis and the type of intervention.

**Figure 4:**
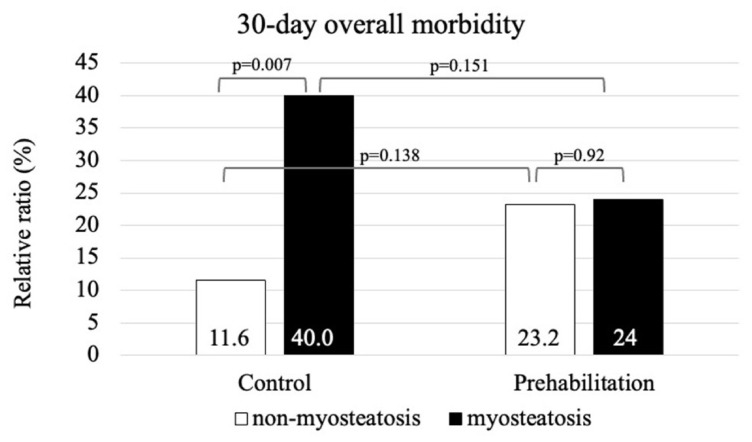
The 30-day overall morbidity. Cases are stratified by myosteatosis and the type of intervention.

Details of the subgroup analyses are provided in Supplementary File S3. Within the ERAS (control) cohort, myosteatosis was associated with significantly higher overall morbidity at both 7 days (7% vs. 28% in non-myosteatotic and myosteatotic patients; *p* = 0.002) and 30 days (11.6% vs. 40%; *p* = 0.007) (Figure 3). In contrast, within the prehabilitation cohort, morbidity did not differ between non-myosteatotic and myosteatotic patients at either 7 days (17.9% vs. 10%; *p* = 0.247) or 30 days (23.2% vs. 24%; *p* = 0.924) (Figure 4). Consistent with these findings, prehabilitation was associated with a reduction in 7-day morbidity among patients with myosteatosis, compared with ERAS alone (*p* = 0.045), whereas the corresponding difference at 30 days did not reach statistical significance (*p* = 0.151). No statistically significant effect of prehabilitation was observed within the non-myosteatotic subgroup (7 days: *p* = 0.112; 30 days: *p* = 0.138).

In BMI-stratified analyses, 30-day mortality differed between patients with BMI < 30 and those with BMI ≥ 30 in the control cohort (0% vs. 9.1%; *p* = 0.03). Conversely, within the prehabilitation cohort, higher BMI was associated with increased overall morbidity at 7 days (8.0% vs. 20.8%; *p* = 0.03) and 30 days (12.5% vs. 37.5%; *p* = 0.001), without a corresponding difference in mortality (0% vs. 2.1%; *p* = 0.353). Finally, frailty emerged as a significant marker of severe postoperative complications (Clavien–Dindo grade ≥ 3) at both 7 days (2.2% vs. 0% vs. 16.0%; *p* = 0.018) and 30 days (5.6% vs. 2.6% vs. 12.7%; *p* = 0.028), independent of intervention allocation.

## 4. Discussion

The ERAS (enhanced recovery after surgery) pathway is a standardized, evidence-based perioperative protocol designed to reduce surgical stress and has been widely adopted internationally. ERAS has been regarded as a gold-standard approach, supported by data demonstrating reductions in postoperative morbidity, mortality, and length of hospital stay [10,11]. Nevertheless, a clinically important subset of patients remains insufficiently resilient to tolerate major surgery, even within an optimal ERAS framework, and may fail to regain postoperative independence. In this context, prehabilitation has emerged as a proposed strategy to enhance physiological reserve before surgery. However, prehabilitation programs remain heterogeneous and are still evolving, and consensus recommendations regarding optimal content, intensity, duration and eligible target populations are lacking [20].

The absence of clear guidance is likely driven, at least in part, by the marked variability of prehabilitation interventions. Early unimodal programs aimed to improve a single modifiable domain, whereas contemporary practice increasingly favors tri-modal or multimodal approaches which integrate nutritional optimization, physical training and psychological preparation, often complemented by support for cessation of harmful behaviors. Even within multimodal models, substantial heterogeneity persists, as some protocols prioritize aerobic conditioning, while others emphasize resistance training, flexibility or respiratory and musculoskeletal function [21]. Unsurprisingly, such variation may translate into inconsistent clinical endpoints. Although improvements in preoperative physical function are frequently reported following exercise-based prehabilitation, effects on postoperative morbidity and mortality have been variable across studies [22]. In addition, the appropriate target population for prehabilitation remains uncertain, with no uniform criteria for patient selection [13,23].

In our study, we enrolled 244 patients, constituting one of the largest cohorts reported in this field. Participants received either ERAS alone or tri-modal prehabilitation followed by ERAS. Because current guidelines strongly recommend oral nutritional supplementation when indicated, nutritional assessment and supplementation were offered in both arms [14,15]. Baseline characteristics were statistically comparable between treatment groups. Within the prehabilitation subcohort, functional measures, most notably the 6 min walking distance (6MWD) and incentive spirometry, improved on the day of surgery. However, this between-group difference was no longer evident at the 8-week follow-up. These findings are consistent with prior work, including a trial by Chen et al., in which 6MWD improved preoperatively among prehabilitated patients [24]. Our results also align with a recent meta-analysis encompassing 1961 colorectal patients across 17 studies, reporting that multimodal prehabilitation improved functional capacity predominantly in the preoperative period [25]. Notably, earlier studies have only provided limited data regarding longitudinal changes in respiratory function during prehabilitation.

Reported effects of prehabilitation on postoperative outcomes have been mixed. While some studies have documented reductions in overall morbidity and severe morbidity in unselected colorectal cohorts, multiple systematic reviews and meta-analyses have found no statistically significant differences in overall morbidity or mortality between prehabilitated and non-prehabilitated groups [6,8,11,26,27,28,29,30]. Our results are concordant with the latter, showing no significant differences in overall morbidity, severe morbidity or mortality between groups when applied to a non-selected real-world population. One plausible explanation relates to the central contribution of nutritional optimization. In a large randomized controlled trial frequently cited in this domain, patients at an a priori elevated risk of postoperative mortality (e.g., those undergoing abdomino-perineal resection or living with chronic kidney failure) were excluded, and the control arm received no structured intervention, such as dietary counseling, potentially limiting generalizability to heterogeneous clinical practice [11]. By contrast, in our study, both groups underwent preoperative dietitian-led nutritional assessment and received oral nutritional supplements when indicated. Given recommendations to optimize nutritional status preoperatively, withholding nutritional support from controls was not considered ethically acceptable [31]. Collectively, these considerations suggest that nutritional prehabilitation may represent a strong component of multimodal programs.

Because prehabilitation is resource-intensive, requiring specialized personnel, infrastructure, and equipment, several groups have advocated for a selective approach targeting subgroups most likely to benefit. For example, Rooijen et al. explored preoperative modifiable risk factors that may influence prehabilitation responsiveness [32]. Other investigators have focused on older adults, reporting functional gains in this population [13,33]. In frail patients, Chang et al. observed reductions in postoperative morbidity and length of hospital stay [34]. Quang et al. reported fewer hospital days among patients with a preoperative 6MWD exceeding 431 m [35]. Despite widespread use of 6MWD for selection in many centers, its validity as a screening tool for prehabilitation eligibility remains insufficiently established. In our cohort, neither the mFI-5 frailty score nor 6MWD reliably identified patients who benefitted differentially from ERAS alone versus prehabilitation plus ERAS. Given the absence of significant between-group differences, even with our comparatively large sample, we performed exploratory subgroup analyses, which suggested that imaging-derived parameters, particularly myosteatosis, may signal increased susceptibility to postoperative morbidity and mortality.

Myosteatosis reflects fatty infiltration and degeneration of skeletal muscle, and has been linked to impaired muscle performance, reduced functional reserve, and vulnerability to immobilization. Consistent with this concept, Blackwell et al. reported that compromised muscle mass and muscle quality, including sarcopenia and myosteatosis identified on preoperative imaging, are associated with worse postoperative outcomes [36,37]. These observations support the idea of myosteatosis as a selection marker for prehabilitation. Importantly, implementation would not require additional testing or costs, as preoperative staging CT imaging is routinely performed and can be utilized to quantify myosteatosis. In our cohort, a myosteatosis threshold of 27.5 HU applied to both sexes classified 43% of colorectal cancer cases as myosteatotic. However, given international variation in body composition, different populations may warrant different cut-offs, which could complicate cross-study comparability. To our knowledge, we are the first to describe myosteatosis as a plausible predictor of sequential morbidity and mortality among patients treated with ERAS alone or with adjunct prehabilitation. This observation is supported by a recent meta-analysis of 9203 patients across 10 studies by Chang et al., which found that myosteatosis is associated with poorer survival outcomes among patients undergoing curative colorectal cancer surgery [38].

This study has limitations, including its single-center design, observational nature, and the potential for residual confounding, as well as the absence of long-term follow-up. We further acknowledge that no formal a priori sample size calculation was performed. The ERAS-alone group may be considered to have received a form of unimodal nutritional prehabilitation, as nutritional assessment and supplementation were provided in both study arms for ethical reasons. Although we think that the psychological evaluation was completed in each case to the best of our capabilities, residual inconsistencies might have biased the outcome. Moreover, our subgroup analyses were exploratory and not powered to establish definitive causal associations. Larger, prospective studies are needed to confirm the proposed selection markers. These limitations should be weighed against the strengths of the study, including detailed nutritional and functional characterization and CT-based body composition assessment.

## 5. Conclusions

While trimodal prehabilitation enhances preoperative functional capacity, it does not significantly impact short-term clinical outcomes in the unselected general colorectal surgery population. Myosteatosis assessment via preoperative CT may help identify patients who benefit most from targeted trimodal prehabilitation.

## Figures and Tables

**Table 1:** Distribution of baseline clinical and anthropometrical parameters among included patients, grouped by study intervention.

	ERAS n = 104	Prehabilitation n = 140	*p* Value
**Age; years** (median; IQR (min–max)	69; 11.6 (24–88)	70; 11.2 (25–88)	0.712 ^a^
**Sex; female** (n, %)	48 (46.2%)	68 (48.6%)	0.708 ^b^
**Comorbidities:**			
Previous laparotomy (n, %)	59 (56.7%)	71 (50.7%)	0.352 ^b^
Diabetes mellitus (n, %)	32 (30.8%)	35 (25%)	0.318 ^b^
Essential hypertension (n, %)	55 (52.8%)	69 (49.3%)	0.751^b^
Cerebrovascular disease (n, %)	11 (10.6%)	14 (10%)	0.883 ^b^
Pulmonary disease (n, %)	6 (5.8%)	8 (5.7%)	0.985 ^b^
Kidney disease (n, %)	2 (1.9%)	6 (4.3%)	0.305 ^b^
Liver diseases (n; %)	1 (0.96%)	0 (0%)	0.245 ^b^
Peripheral vascular disease (n, %)	2 (1.9%)	5 (3.6%)	0.446 ^b^
Heart disease (n, %)	15 (14.4%)	29 (20.7%)	0.206 ^b^
Smoking (n, %)	34 (32.7%)	49 (35%)	0.707 ^b^
Alcohol consumer (n, %)	50 (48.1%)	77 (55%)	0.284 ^b^
**Neoadjuvant oncotherapy** (n, %)	20 (19.2%)	24 (17.1%)	0.675 ^b^
**CR-POSSUM score** (median; IQR (min–max)	1.9; 4.3 (0.5–22.5)	1.9; 3.6 (0.3–21.5)	0.750 ^a^
**Regular medication:**			
Anticoagulant drugs (n, %)	11 (10.6%)	24 (17.1%)	0.148 ^b^
Antithrombotic drugs (n, %)	26 (25%)	30 (21.4%)	0.512 ^b^
Systemic corticosteroids (n, %)	5 (4.8%)	8 (5.7%)	0.755 ^b^
**Anthropometric data:**			
BMI; kg/m^2^ (median; IQR (min–max)	27.9; 5.6 (17.8–47.7)	27.9; 5.3 (18.2–43.7)	0.750 ^a^
Body fat percentage (median; IQR (min–max)	32; 11.3 (9.6–65)	34.2; 11.5 (11.9–60.7)	0.474 ^a^
**MUST score** (n, %)			
1	12 (11.5%)	24 (17.1%)	0.199 ^b^
2	41 (39.4%)	62 (44.3%)	
3	48 (46.2)	50 (35.7%)	
**Modified Frailty Index 5 score** (n, %)			
Low (mFI-5 = 0)	27 (26%)	45 (32.1%)	0.119 ^b^
Medium (mFI-5 = 1)	47 (45.2%)	70 (50%)	
Severe (mFI-5 ≥ 2)	30 (28.8%)	25 (17.9%)	
**Sarcopenia** (n, %)	23 (%)	29 (%)	0.378 ^b^
**L3 skeletal muscle index; cm^2^/m^2^**	49; 9	48.3; 9	0.545 ^a^
(median; IQR (min–max)	(23.2–68.2)	(29–74.1)	
**Psoas Index; cm^2^/m^2^**	6.4; 1.6	5.8; 1.9	0.528 ^a^
(median; IQR (min–max)	(2.35–10.05)	(0–11.3)	
**Myosteatosis; Hounsfield Unit**	30.6; 6.8	27.9; 8.2	0.067 ^a^
(median; IQR (min–max)	(14.2–43.9)	(12.7–57.6)	

^a^: Mann–Whitney U test, ^b^: Chi-square test.

**Table 2:** Distribution of operation and pathological parameters among included patients, grouped by study intervention.

	ERAS n = 104	Prehabilitation n = 140	*p* Value
**Tumor localisation (n,%)**			
Colon	43 (41.4%)	67 (47.9%)	0.590 ^b^
Rectum	59 (56.7%)	71 (50.7%)	
Other	2 (1.9%)	2 (1.4%)	
**Operation type (n,%)**			
Right hemicolectomy	29 (27.9%)	37 (26.5%)	0.381 ^b^
Extended right hemicolectomy	4 (3.9%)	8 (5.7%)	
Transversum resection	2 (1.9%)	0	
Splenic flexure resection	0	1 (0.7%)	
Left hemicolectomy	4 (3.9%)	4 (2.9%)	
Sigma resection	5 (4.8%)	17 (12.1%)	
Anterior resection	38 (36.5%)	42 (30%)	
Low anterior resection	11 (10.6%)	17 (12.1%)	
Abdominoperineal resection	9 (8.6%)	12 (8.6%)	
Colorectal reconstruction	2 (1.9%)	2 (1.4%)	
**Access (n,%)**			
Laparoscopic	88 (84.6%)	122 (87.1%)	0.764 ^b^
Open	11 (10.6%)	11 (7.9%)	
Conversion to open surgery	5 (4.8%)	7 (5%)	
**Operation lenght; min** (median; IQR (min–max)	170; 73 (55–445)	164; 80.1 (60–390)	0.844 ^a^
**Stoma formation** (n, %)	22 (21.1%)	33 (23.6%)	0.655 ^b^
**Stoma type (n, %)**			
Ileostomy	10 (9.6%)	17 (12.1%)	0.792 ^b^
Transversostomy	1 (1%)	0	
Biluminal sigmoideostomy	1 (1%)	2 (1.4%)	
Hartmann’s stoma	1 (1%)	3 (2.1%)	
End colostomy for APR	9 (8.7%)	11 (7.9%)	
No stoma	82 (78.7%)	107 (76.5%)	
**Tumor characteristics**			
Malignant (n, %)	77 (74%)	106 (75.7%)	0.765 ^b^
**Pathological TNM stage (n,%)**			
T1	7 (9.1%)	8 (7.5%)	0.940 ^b^
T2	15 (19.5%)	24 (22.6%)	
T3	43 (55.8%)	59 (55.7%)	
T4	12 (15.6%)	15 (14.2%)	
N0	45 (58.4%)	70 (66%)	0.027 ^b^
N1	18 (23.4%)	30 (28.3%)	
N2	14 (18.2%)	6 (5.7%)	
M0	70 (90.9%)	95 (89.6%)	0.773 ^b^
M1	7 (9.1%)	11 (10.4%)	
**Histopathological grade (n,%)**			
na	35 (33.6%)	43 (30.7%)	0.309 ^b^
1	0	3 (2.1%)	
2	65 (62.5%)	87 (62.2%)	
3	4 (3.9%)	7 (5%)	

^a^: Mann–Whitney U test, ^b^: Chi-square test.

**Table 3:** Functional outcomes among included patients, grouped by study intervention.

	ERAS n = 104	Prehabilitation n = 140	*p* Value
**6 min walking distance (6MWD)** (median; IQR (min–max)			
Baseline (m)	195; 112 (22–708)	194; 124 (9–648)	0.687 ^a^
At time of surgery (m)	210; 128 (23–700)	256; 152.4 (0–729)	0.099 ^a^
Time of surgery; % from baseline	106; 24.8 (63–206)	121; 52.8 (0–376)	<0.001 ^a^
Change by time of surgery; absolute value (m)	18; 53.1 (−129–204)	52; 84.9 (−367–339)	<0.001 ^a^
8 weeks postoperatively; (m)	255; 129.6 (34–705)	255; 133.6 (12–650)	0.392 ^a^
8 weeks postoperatively; % from baseline	107.4; 48.7 (0–233)	101.7; 67.7 (0–368)	0.876 ^a^
Change by 8 weeks postoperatively; absolute value (m)	34; 98.3 (−297–256)	31; 78.2 (−177–272)	0.518 ^a^
**Incentive spirometry-forced vital capacity** (median; IQR (min–max)			
Baseline (mL)	2500; 971.9 (1000–5000)	2250; 1103.5 (400–6490)	0.322 ^a^
Time of surgery (mL)	2500; 1037.1 (750–5000)	2650; 1047.3 (1250–5000)	0.228 ^a^
Time of surgery; % from baseline	100; 18.8 (60–167)	114.3; 39.4 (0–300)	0.001 ^a^
Change by time of surgery; absolute value (mL)	0; 410 (−500–2500)	0; 497 (−1490–2500)	0.024 ^a^
8 weeks postoperatively; (mL)	3000; 1100 (750–5000)	2625; 1030.7 (1500–5000)	0.501 ^a^
8 weeks postoperatively; % from baseline	111.3; 21 (75–175)	112.9; 36.3 (0–275)	0.552 ^a^
Change by 8 weeks postoperatively; absolute value (mL)	250; 445 (−500–1500)	250; 716.5 (−3490–2250)	0.912 ^a^
**Objective spirometry-forced vital capacity** (median; IQR (min–max)			
Baseline (mL)	3300; 1183.8 (1250–6880)	3070; 1069.7 (400–5870)	0.156 ^a^
Time of surgery (mL)	3245; 1072 (1670–7080)	3360; 1156.2 (1180–6511)	0.607 ^a^
Time of surgery; % from baseline	99.3; 9.9 (72–130)	100.4; 26.5 (0–237)	0.872 ^a^
Change by time of surgery; absolute value	−20; 306.1 (−1130–810)	20; 666 (−1280–3761)	0.878 ^a^
8 weeks postoperatively; (mL)	3470; 1107.8 (1820–6380)	3280; 926.3 (1570–5720)	0.120 ^a^
8 weeks postoperatively; % from baseline	100.2; 13.2 (72–166)	100.3; 17.5 (86–161)	0.641 ^a^
Change by 8 weeks postoperatively; absolute value (mL)	10; 368.5 (−1420–890)	10; 481.6 (−550–1500)	0.578 ^a^

^a^: Mann–Whitney U test.

**Table 4:** Postoperative outcome for patients allocated to prehabilitation or ERAS group.

	ERAS n = 104	Prehabilitation n = 140	*p* Value
**Morbidity**			
7-day overall (n, %)	16 (15.4%)	17 (12.1%)	0.464 ^b^
30-day overall (n, %)	23 (22.1%)	31 (22.1%)	0.996 ^b^
Clavien–Dindo ≥ grade-3 (7 days) (n, %)	3 (2.9%)	5 (3.6%)	1 ^c^
Clavien–Dindo ≥ grade-3 (30 days) (n, %)	5 (4.8%)	9 (6.4%)	0.782 ^c^
**Mortality**			
7-day (n, %)	2 (1.9%)	0 (0%)	0.181 ^c^
30-day (n, %)	3 (2.9%)	1 (0.7%)	0.315 ^c^
**Other outcomes**			
Postoperative ICU days (median; IQR (min–max)	0; 0.65 (0–4)	0; 0.42 (0–2)	0.304 ^a^
Length of hospital stay; days (median; IQR (min–max)	7; 4.3 (4–42)	7; 5.7 (3–47)	0.065 ^a^
Readmission within 30 days (n, %)	7 (6.7%)	10 (7.1%)	0.9 ^b^

^a^: Mann–Whitney U test, ^b^: Chi-square test, ^c^: Fisher exact test.

**Table 5:** Univariable and multivariable logistic regression models for overall postoperative morbidity at postoperative, at day 7 (**A**) and day 30 (**B**).

(**A**)						
	**Univariate Analysis**			**Multivariate Analysis**		
	**Odds Ratio**	**95% CI**	***p* Value**	**Odds Ratio**	**95% CI**	***p* Value**
**Age**	0.99	0.96–1.01	0.30	0.97	0.92–1.03	0.30
**Male sex**	1.10	0.53–2.30	0.79	0.68	0.20–2.30	0.53
**Prehabilitation performed**	0.76	0.36–1.59	0.46	0.40	0.12–1.28	0.12
**BMI**	0.98	0.92–1.06	0.67	n.a.		
**CR-POSSUM score**	0.95	0.84–1.07	0.41	n.a.		
**6MWD**	1.00	1.00–1.00	0.18	n.a.		
**FVC**	1.00	1.00–1.00	0.22	n.a.		
**MUST score**	0.74	0.44–1.25	0.26	0.67	0.30–1.48	0.31
**mFI-5**	1.01	0.57–1.78	0.97	0.69	0.31–1.58	0.38
**Sarcopenia present**	0.84	0.28–2.50	0.75	0.96	0.29–3.15	0.9
**Myosteatosis present**	0.99	0.93–1.05	0.68	1.11	0.93–1.34	0.25
(**B**)						
	**Univariate Analysis**			**Multivariate Analysis**		
	**Odds Ratio**	**95% CI**	***p* Value**	**Odds Ratio**	**95% CI**	***p* Value**
**Age**	0.99	0.97–1.02	0.67	0.95	0.90–1.00	0.03
**Male sex**	0.88	0.48–1.61	0.68	0.49	0.18–1.32	0.15
**Prehabilitation performed**	1.00	0.54–1.84	0.99	0.58	0.22–1.52	0.27
**BMI**	1.04	0.98–1.10	0.22	n.a.		
**CR-POSSUM score**	1.08	0.97–1.21	0.15	n.a.		
**6MWD**	1.00	1.00–1.00	0.93	n.a.		
**FVC**	1.00	1.00–1.00	0.11	n.a.		
**MUST score**	1.03	0.67–1.61	0.88	1.16	0.59–2.28	0.65
**mFI-5**	1.27	0.81–1.98	0.29	1.10	0.60–2.03	0.75
**Sarcopenia present**	0.76	0.32–1.83	0.54	0.67	0.26–1.75	0.41
**Myosteatosis present**	0.99	0.94–1.03	0.61	1.18	1.01–1.38	0.03

## Data Availability

The data presented in this study are available on request from the corresponding author due to national ethical restrictions.

## Supplementary Materials

The following supporting information can be downloaded at: https://www.mdpi.com/article/10.3390/nu18091369/s1, File S1: Dietetic questionnare; File S2: Study intervention protocol; File S3: Subgroup analysis within the ERAS and prehabilitation subcohorts, grouped according to study outcomes.

## Abbreviations

6MWD	6 min walking distance
BMI	body mass index
CR-POSSUM	ColoRectal Physiological and Operative Severety Score
ERAS	Enhanced Recovery after Surgery
FVC	forced vital capacity
HU	Hounsfield Unite
L3SMI	lumbal 3 skeletal muscle index
LOS	length of stay
MUST	Malnutrition Universal Screening Tool
mFI-5	modified 5-item frailty index
TAMIS	Transanal Minimally Invasive Surgery

## References

[1] Observatory WGC: Estimated Number of Deaths Worldwide, Both Sexes, All Ages (Graph). https://gco.iarc.fr/.

[2] Byrne B.E., Mamidanna R., Vincent C.A., Faiz O. (2013). Population-based cohort study comparing 30- and 90-day institutional mortality rates after colorectal surgery. Br. J. Surg..

[3] Alves A., Panis Y., Mathieu P., Mantion G., Kwiatkowski F., Slim K., Association Francaise de Chirurgie (2005). Postoperative mortality and morbidity in French patients undergoing colorectal surgery: Results of a prospective multicenter study. Arch. Surg..

[4] Hinojosa M.W., Konyalian V.R., Murrell Z.A., Varela J.E., Stamos M.J., Nguyen N.T. (2007). Outcomes of right and left colectomy at academic centers. Am. Surg..

[5] Jones L.W., Eves N.D., Haykowsky M., Freedland S.J., Mackey J.R. (2009). Exercise intolerance in cancer and the role of exercise therapy to reverse dysfunction. Lancet Oncol..

[6] Gustafsson U.O., Scott M.J., Hubner M., Nygren J., Demartines N., Francis N., Rockall T.A., Young-Fadok T.M., Hill A.G., Soop M. (2019). Guidelines for Perioperative Care in Elective Colorectal Surgery: Enhanced Recovery After Surgery (ERAS^®^) Society Recommendations: 2018. World J. Surg..

[7] Wind J., Hofland J., Preckel B., Hollmann M.W., Bossuyt P.M., Gouma D.J., van Berge Henegouwen M.I., Fuhring J.W., Dejong C.H., van Dam R.M. (2006). Perioperative strategy in colonic surgery; LAparoscopy and/or FAst track multimodal management versus standard care (LAFA trial). BMC Surg..

[8] Gustafsson U.O., Scott M.J., Schwenk W., Demartines N., Roulin D., Francis N., McNaught C.E., MacFie J., Liberman A.S., Soop M. (2012). Guidelines for perioperative care in elective colonic surgery: Enhanced Recovery After Surgery (ERAS^®^) Society recommendations. Clin. Nutr..

[9] Li C., Carli F., Lee L., Charlebois P., Stein B., Liberman A.S., Kaneva P., Augustin B., Wongyingsinn M., Gamsa A. (2013). Impact of a trimodal prehabilitation program on functional recovery after colorectal cancer surgery: A pilot study. Surg. Endosc..

[10] Keller D.S., Curtis N., Burt H.A., Ammirati C.A., Collings A.T., Polk H.C., Carrano F.M., Antoniou S.A., Hanna N., Piotet L.M. (2024). EAES/SAGES evidence-based recommendations and expert consensus on optimization of perioperative care in older adults. Surg. Endosc..

[11] Molenaar C.J.L., Minnella E.M., Coca-Martinez M., Ten Cate D.W.G., Regis M., Awasthi R., Martinez-Palli G., Lopez-Baamonde M., Sebio-Garcia R., Feo C.V. (2023). Effect of Multimodal Prehabilitation on Reducing Postoperative Complications and Enhancing Functional Capacity Following Colorectal Cancer Surgery: The PREHAB Randomized Clinical Trial. JAMA Surg..

[12] Fulop A., Lakatos L., Susztak N., Szijarto A., Banky B. (2021). The effect of trimodal prehabilitation on the physical and psychological health of patients undergoing colorectal surgery: A randomised clinical trial. Anaesthesia.

[13] Looijaard S., Slee-Valentijn M.S., Otten R.H.J., Maier A.B. (2018). Physical and Nutritional Prehabilitation in Older Patients with Colorectal Carcinoma: A Systematic Review. J. Geriatr. Phys. Ther..

[14] Weimann A., Braga M., Carli F., Higashiguchi T., Hubner M., Klek S., Laviano A., Ljungqvist O., Lobo D.N., Martindale R. (2017). ESPEN guideline: Clinical nutrition in surgery. Clin. Nutr..

[15] Weimann A., Wobith M. (2024). ESPEN Guidelines on Clinical nutrition in surgery-Special issues to be revisited. Eur. J. Surg. Oncol..

[16] Subramaniam S., Aalberg J.J., Soriano R.P., Divino C.M. (2018). New 5-Factor Modified Frailty Index Using American College of Surgeons NSQIP Data. J. Am. Coll. Surg..

[17] Tekkis P.P., Prytherch D.R., Kocher H.M., Senapati A., Poloniecki J.D., Stamatakis J.D., Windsor A.C. (2004). Development of a dedicated risk-adjustment scoring system for colorectal surgery (colorectal POSSUM). Br. J. Surg..

[18] Yajima T., Arao M., Yajima K. (2022). Psoas muscle index and psoas muscle density as predictors of mortality in patients undergoing hemodialysis. Sci. Rep..

[19] Yoon J.K., Lee S., Kim K.W., Lee J.E., Hwang J.A., Park T., Lee J. (2021). Reference Values for Skeletal Muscle Mass at the Third Lumbar Vertebral Level Measured by Computed Tomography in a Healthy Korean Population. Endocrinol. Metab..

[20] Gustafsson U., Rockall T., Wexner S., How K., Emile S., Marchuk A., Fawcett W., Sioson M., Riedel M., Chahal R. (2025). Guidelines for perioperative care in elective colorectal surgery: Enhanced Recovery After Surgery (ERAS) Society recommendations 2025. Surgery.

[21] Molenaar C.J., van Rooijen S.J., Fokkenrood H.J., Roumen R.M., Janssen L., Slooter G.D. (2023). Prehabilitation versus no prehabilitation to improve functional capacity, reduce postoperative complications and improve quality of life in colorectal cancer surgery. Cochrane Database Syst. Rev..

[22] Garoufalia Z., Emile S.H., Meknarit S., Gefen R., Horesh N., Zhou P., Aeschbacher P., Strassmann V., Wexner S.D. (2024). A systematic review and meta-analysis of high-quality randomized controlled trials on the role of prehabilitation programs in colorectal surgery. Surgery.

[23] She K.Y., Huang L., Zhang H.T., Gao Y., Yao K.R., Luo Q., Tang X., Li L., Zhao L., Wang Z.H. (2024). Effect of prehabilitation on postoperative outcomes in the frail older people: A systematic review and meta-analysis. Geriatr. Nurs..

[24] Chen B.P., Awasthi R., Sweet S.N., Minnella E.M., Bergdahl A., Santa Mina D., Carli F., Scheede-Bergdahl C. (2017). Four-week prehabilitation program is sufficient to modify exercise behaviors and improve preoperative functional walking capacity in patients with colorectal cancer. Support. Care Cancer.

[25] Zhou L., Li H., Zhang Z., Wang L. (2024). Effects of multimodal prehabilitation and exercise prehabilitation on patients undergoing colorectal surgery: A systematic review and meta-analysis of randomised controlled trials. J. Glob. Health.

[26] Banky B., Lakatos L., Rozman P., Szijarto A. (2023). Surgery of the colorectal polyps and early stage cancer-Expected standards. Magy. Seb..

[27] Sabajo C.R., Ten Cate D.W.G., Heijmans M.H.M., Koot C.T.G., van Leeuwen L.V.L., Slooter G.D. (2024). Prehabilitation in colorectal cancer surgery improves outcome and reduces hospital costs. Eur. J. Surg. Oncol..

[28] Hogeweg-Raaijmakers J.G.M., Bossema E.R., Teeuwen P.H.E., Fusers A., van Vliet M., Wegdam J.A. (2022). A prehabilitation program for patients undergoing elective resection of a colorectal carcinoma: Effects on the postoperative hospital stay and complication burden. Ned. Tijdschr. Geneeskd..

[29] Wee I.J.Y., Seow-En I., Chok A.Y., Sim E., Koo C.H., Lin W., Meihuan C., Tan E.K. (2024). Postoperative outcomes after prehabilitation for colorectal cancer patients undergoing surgery: A systematic review and meta-analysis of randomized and nonrandomized studies. Ann. Coloproctol..

[30] Lambert J., Hayes L., Keegan T., Subar D., Gaffney C. (2021). Response to the Comment on “The Impact of Prehabilitation on Patient Outcomes in Hepatobiliary, Colorectal and Upper Gastrointestinal Cancer Surgery: A PRISMA-Accordant Meta-analysis”. Ann. Surg..

[31] Hijazi Y., Gondal U., Aziz O. (2017). A systematic review of prehabilitation programs in abdominal cancer surgery. Int. J. Surg..

[32] van Rooijen S., Carli F., Dalton S.O., Johansen C., Dieleman J., Roumen R., Slooter G. (2017). Preoperative modifiable risk factors in colorectal surgery: An observational cohort study identifying the possible value of prehabilitation. Acta Oncol..

[33] Bruns E.R., van den Heuvel B., Buskens C.J., van Duijvendijk P., Festen S., Wassenaar E.B., van der Zaag E.S., Bemelman W.A., van Munster B.C. (2016). The effects of physical prehabilitation in elderly patients undergoing colorectal surgery: A systematic review. Color. Dis..

[34] Chang M.C., Choo Y.J., Kim S. (2023). Effect of prehabilitation on patients with frailty undergoing colorectal cancer surgery: A systematic review and meta-analysis. Ann. Surg. Treat. Res..

[35] Le Quang A.T., Carli F., Prince F. (2023). Is preoperative physical function testing predictive of length of stay in patients with colorectal cancer? A retrospective study. Eur. J. Surg. Oncol..

[36] Jung H.N., Cho Y.K., Kim H.S., Kim E.H., Lee M.J., Lee W.J., Kim H.K., Jung C.H. (2023). Association between hypertension and myosteatosis evaluated by abdominal computed tomography. Hypertens. Res..

[37] Blackwell J.E.M., Herrod P.J.J., Doleman B., Boyd-Carson H., Dolan D., Wheldon L., Brown S.R., Banerjea A., Moug S., Lund J.N. (2023). CT-derived measures of muscle quantity and quality predict poorer outcomes from elective colorectal surgery: A UK multicentre retrospective cohort study. Tech. Coloproctol..

[38] Chang Y.Y., Cheng B. (2024). Prognostic impact of myosteatosis in patients with colorectal cancer undergoing curative surgery: An updated systematic review and meta-analysis. Front. Oncol..

