# Supplementary material for: Identifying Patients with Colorectal Cancer Likely to Benefit from a Trimodal Prehabilitation Prior to Surgery
Source: Nutrients. 2026 Apr 27;18(9):1369. doi: 10.3390/nu18091369 (PMC13164859; doi:10.3390/nu18091369)
Supplement: Supplementary file 1 [file nutrients-18-01369-s001.zip › Prehab-study-supplementary-File S1.pdf]

# Dietetic Questionnaire

## Nutritional History

**Do you have any food allergies?**

☐ Yes
☐ No
Food allergy: ___________________________________

**Do you take vitamin C?**

☐ Yes
☐ No

**Are there any foods that cause symptoms?**

☐ Yes
☐ No
Food causing symptoms: ___________________________________

**How many times do you eat per day?** ___________________________________

**How many times per week do you consume meat?** ___________________________________

**How many times per week do you consume processed meat products?** ____________________________

**What type of meat do you consume?**

☐ Red meat
☐ Poultry
☐ Fish

**Do you consume milk or dairy products (cottage cheese, cheese, yogurt)?**

☐ Yes
☐ No

**How much milk do you consume per day?**

☐ 0.5 liters or less
☐ More than 0.5 liters

**How many times per week do you consume dairy products?** ___________________________________

**How many eggs do you consume per week?** ___________________________________

**Do you use animal fat?**

☐ Yes
☐ No

**Do you consume vegetables daily?**

☐ Yes
☐ No

**Amount of vegetable consumption:**

☐ Less than 300 g/day
☐ Between 300 and 500 g/day
☐ More than 500 g/day

**Do you consume fruit daily?**

☐ Yes
☐ No

**Amount of fruit consumption:**

- ☐ Less than 300 g/day
- ☐ Between 300 and 500 g/day
- ☐ More than 500 g/day

**Do you consume snack foods/sweets (chocolate, candy, pastries, salty sticks, chips, nuts/seeds)?**

- ☐ Yes
- ☐ No

**How many times per week do you consume snack foods/sweets?** ________________________________

**How much sugar do you use daily for sweetening?** __________________________________

**Do you use artificial sweeteners?**

- ☐ Yes
- ☐ No

**Do you consume any nutritional supplement/formula?**

- ☐ Yes
- ☐ No

**What nutritional supplement/formula do you consume?** __________________________________

## Medical History

**Diabetes**

- ☐ None
- ☐ Diet
- ☐ Tablets
- ☐ Insulin
- ☐ No data

**Chronic kidney failure**

- ☐ None
- ☐ Medication
- ☐ Dialysis
- ☐ Transplant
- ☐ No data

**Liver disease**

- ☐ None
- ☐ Hepatitis
- ☐ Cirrhosis (compensated)
- ☐ Cirrhosis (vascular decompensation)
- ☐ Cirrhosis (parenchymal decompensation)
- ☐ Other __________________________________________

## Lifestyle

**Alcohol: How often do you drink?**

- ☐ Do not drink
- ☐ Rarely
- ☐ Every day
- ☐ Stopped less than 1 year ago
- ☐ Stopped more than 1 year ago
- ☐ No data

## Medication History

**Steroids**

☐ None
☐ Local (budesonide, etc.)
☐ Systemic

**Did the patient receive neoadjuvant chemotherapy?**

☐ Yes
☐ No

**Was neoadjuvant irradiation performed?**

☐ Yes
☐ No

## Risk Screening

**Body weight (kg)** ________________________________

**Height (cm)** ________________________________

**BMI (kg/m²)** ________________________________

**Body weight change in the last 3 months (kg)** ________________________________

**Body fat %** ________________________________

**MUST risk**

☐ Low
☐ Medium
☐ High

## Nutritional Supplement

**Recommended nutritional supplement:**

☐ Nutridrink 2–3 × 1 bottle / day
☐ Nurtidrink Max 1 bottle / day
☐ Kabi-Glutamin 1–2 × 1 sachet / day
☐ Fresubin Hepa 2 × 1 bottle / day
☐ Diasip 2 × 1 bottle / day
☐ Fortimel 2 × 1 bottle / day
☐ Other

Other supplement: ________________________________
