# Supplementary material for: Identifying Patients with Colorectal Cancer Likely to Benefit from a Trimodal Prehabilitation Prior to Surgery
Source: Nutrients. 2026 Apr 27;18(9):1369. doi: 10.3390/nu18091369 (PMC13164859; doi:10.3390/nu18091369)
Supplement: Supplementary file 1 [file nutrients-18-01369-s001.zip › Prehab-study-supplementary-File S2.pdf]

INTRODUCTION OF A PREHABILITATION PROGRAM IN THE COMPLEX SURGICAL MANAGEMENT OF GASTROINTESTINAL TUMORS

Balázs Bánky, MD, PhD

Szent Borbála Hospital, Tatabánya

Department of Surgery

András Fülöp, MD, PhD

Semmelweis University, Budapest

1st Department of Surgery

Tatabánya, 2017

PATIENT INFORMATION BOOKLET
ACTIVITY LOG

TABLE OF CONTENTS

- 1. Patient information
- 2. Exercise program
- 3. Nutrition program
- 4. Psychological and mental preparation program
- 5. Activity log
- Further information - contacts

PLEASE BRING THIS BOOKLET WITH YOU FOR ALL HOSPITAL VISITS RELATED TO YOUR SURGERY!

RECORD OF APPOINTMENT DATES

**ERAS outpatient clinic**	____ year ____ month ____ day
Physiotherapy / baseline assessment	____ year ____ month ____ day
Physiotherapy (sessions)	____ month/____ day ____ month/____ day<br>____ month/____ day ____ month/____ day
Surgery	Admission: ____ year ____ month ____ day<br>Discharge: ____ year ____ month ____ day
8-week follow-up	____ year ____ month ____ day
Patient ID	□ □ □ □ □ □ □ □ □ □ □ □

1. PATIENT INFORMATION

In Hungary, including Tatabánya and its catchment area, cancer is the second most common cause of death. Based on current knowledge, the most effective treatment for malignant diseases is surgical removal of the tumor.

After surgery, the length of recovery and the speed of regaining strength are strongly influenced by your general condition, exercise tolerance, the strength of your respiratory muscles, and your nutritional status.

At the Department of Surgery of Szent Borbála Hospital, as a leading oncological surgery center, we introduced a new, complex care pathway from 2014 that has already been tested in Western Europe but is still only sporadically used in Hungary. This is the so-called ERAS program (ERAS = Enhanced Recovery After Surgery). It is a 20-element protocol covering preoperative preparation, intraoperative management, and postoperative care. Since introducing it, the rate of complications after major abdominal cancer surgery at our department has decreased substantially. As a result, compared with previous practice, our patients can usually go home recovered 2-3 days earlier.

Despite these demonstrable improvements, major surgery still carries considerable risk and a relatively high complication rate. This is due not only to the 'wasting' effect of the underlying malignancy, but also to the extensive and demanding operation, which places a significant burden on the body.

In recent years, a new concept has emerged based on proposals from several North American and Western European surgical centers. This concept is called surgical PREHABILITATION. The essence of the method is a structured, organized program lasting 3-6 weeks to prepare patients consciously for major abdominal surgery, which can fundamentally improve the chances of recovery.

If you consent, you will take part in a clinical study comparing the effectiveness of different preoperative modules, including the ERAS and PREHABILITATION programs.

THANK YOU FOR YOUR COOPERATION!

2. EXERCISE PROGRAM

The protocol below includes a few short muscle-strengthening exercises as well as nutritional and lifestyle advice. The aim is to improve your endurance and physical and mental condition, thereby speeding up postoperative recovery. The program lasts 4 weeks. The exercises are easy to perform at home. By following the instructions below, we can help you enter surgery in the best possible condition, spend as little time in hospital as possible afterwards, and return to your family sooner.

Below you will find the prescribed movement and breathing exercises with recommended daily repetitions and illustrative figures (source: http://www.szimpatika.hu/cikkek/682/legzotorna). Please tick the activity log once you have completed an item.

1. Breathing exercises

Please perform the exercises preferably once per day: during weeks 1-2 for 10 minutes, and during weeks 3-4 for 15 minutes, as follows:

1. Stand with your feet slightly apart, shoulders relaxed - exhale. Pull your shoulders back, bring the shoulder blades together - inhale.

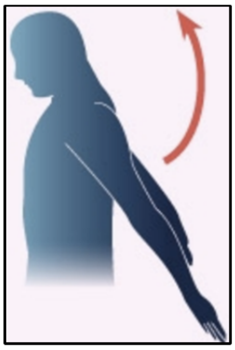

2. Arms alongside the body - exhale. Raise your right arm straight up next to your ear while drawing the left arm back alongside your body - inhale. Then raise the left arm and draw the right arm back.

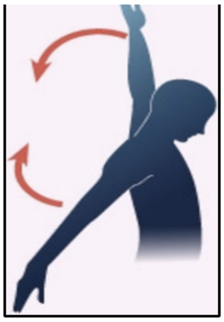

Additional breathing exercises (optional):

3. Right arm alongside the body, left arm crossed in front of you (touching the right hip) - exhale. Raise the left arm diagonally upward - inhale. Repeat with the other arm.

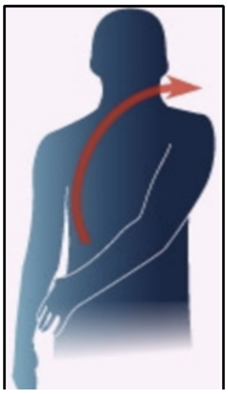

4. Interlace your fingers behind your neck, pull your elbows in front of your face - exhale. Pull your elbows back and lift your chest - inhale.

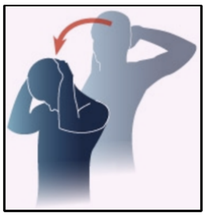

5. Place your hand on your abdomen. During exhalation your abdomen draws in; during inhalation it rises forward.

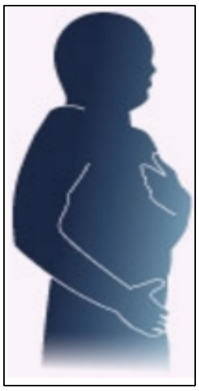

6. Bend your arms, place your hands on your shoulders, then press both elbows toward your chest - exhale. Raise your elbows up to shoulder height - inhale.

7. Same starting position as above, but raise your elbows forward and then to the side - inhale. Lower your elbows back alongside your torso - exhale. (Your elbows trace a circle during the movement.)

8. Interlace your hands in front - exhale. Raise your clasped hands above your head - inhale.

9. Place your hands on your shoulders - exhale. Raise your bent right arm next to your ear and lean to the left - inhale. Repeat to the other side.

2. Spirometer (breathing trainer)

Ideally, you should use the device every hour. If this is not feasible, use it at least 4-5 times per day in the instructed way, at the preset intensity.

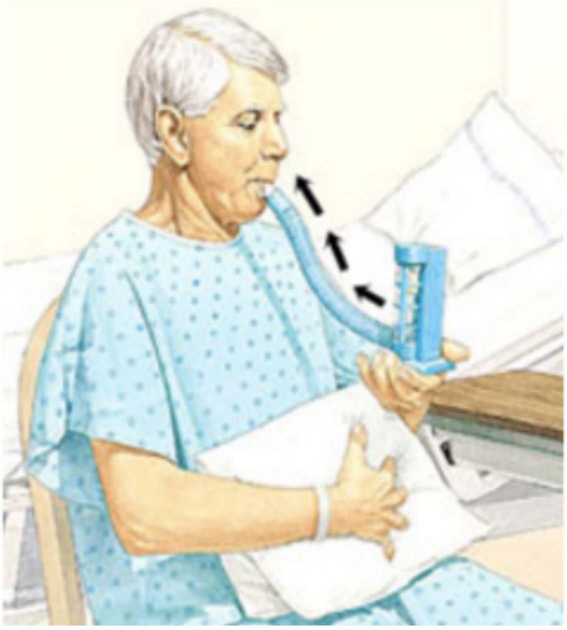

After a slow exhalation, take a deep, prolonged inhalation. Repeat the exercise 10 times per session. (Source: http://www.activeforever.com)

3. Daily walk

Walk for at least 30 minutes per day at a moderate pace. If possible, measure the number of steps using a pedometer/step counter. It is important that you do not record your usual everyday activity; instead, add the daily walk as an extra planned task.

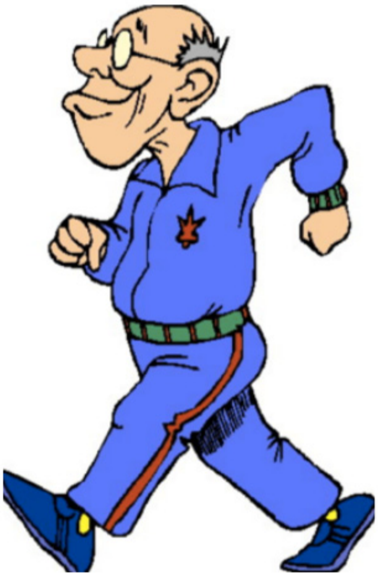

3. NUTRITION PROGRAM

Below you will find dietary advice and recommendations regarding the consumption of required nutritional supplements. Please tick the activity log if you consumed any supplement.

Dietary advice

Low-fat diet: Avoid high-fat meats and meat products. Choose chicken, turkey and fish, preferably steamed/poached, and avoid frying in fat and grilling. Processed meats are not recommended due to their high salt and preservative content. Smoked products should be avoided.

Mild seasoning: Hot paprika, black pepper, chili, horseradish and mustard are not recommended. Instead, use green herbs and mild spices such as lemon balm, thyme, marjoram, caraway, lovage, bay leaf, rosemary, cloves, vanilla, celery, parsley, sweet paprika, oregano.

Avoid coarse fiber and gas-forming foods: Fruit and vegetable peels and seeds can cause unpleasant symptoms such as bloating and cramping. Puree and strain foods so that peel fragments do not remain (e.g., peas, green beans, vegetable purees). Avoid difficult-to-digest, high-fiber vegetables (e.g., cabbage family, onion family, green peppers), as well as corn and dried legumes. Some essential oils may also increase gas (e.g., cauliflower, kohlrabi, onions and cabbage family). To help prevent bloating, eat 5-6 smaller meals per day at regular times and avoid large portions.

Preventing and treating malnutrition: To prevent or treat malnutrition, eating several times per day is recommended.

Oral nutritional supplements: On your physician's advice, to help maintain optimal body weight we recommend special oral nutritional supplements. These are available as ready-to-drink products and as powders. Powders are often used to enrich/fortify meals (individualized to the patient's condition).

Micronutrients: It is important to replace vitamins, antioxidants and minerals.

1. Nutritional supplements

Recommended supplements (to be filled in by your care team):

Supplement	Dose	Route / form
1.) ______________________________ ____	______________________________ ____	______________________________ ____
2.) ______________________________ ____	______________________________ ____	______________________________ ____
3.) ______________________________ ____	______________________________ ____	______________________________ ____

4. PSYCHOLOGICAL AND MENTAL PREPARATION PROGRAM

1. Smoking

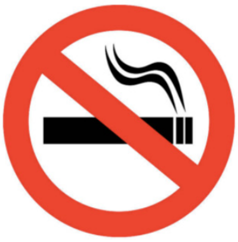

Smoking plays an important role in the development of abnormal conditions of the lungs and the cardiovascular system. Therefore, to improve your physical capacity, complete cessation of smoking is recommended.

During the preparation program and in the postoperative period, stopping smoking is strongly recommended. If you are unable to quit completely, try to reduce your daily amount (for example, cut it by half) to mitigate the harmful effects.

2. Alcohol consumption

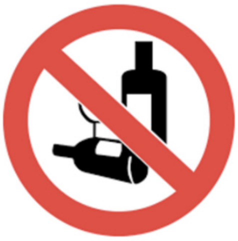

Regular and high-volume alcohol consumption harms the body in many ways. Alcohol, among other effects, damages the liver and pancreas, impairs the nervous and immune systems, worsens cardiac function, and inhibits blood clotting. As a result, excessive alcohol consumption increases the rate of postoperative complications. Therefore, in the period before surgery, reducing alcohol intake is recommended.

During the preparation program and in the postoperative period, it is recommended to reduce alcohol consumption. Try to decrease the quantities you drink and discontinue if possible.

3. Anxiety - fear of surgery - relaxation techniques

It is completely natural to feel worried before undergoing an operation. However, it is very important that fear of surgery and anxiety do not reach a pathological level before the operation. Excessive anxiety can cause unpleasant symptoms such as a sensation of palpitations, an increased heart rate, arrhythmia, high blood pressure, heartburn, nausea, sleep disturbances, and a heightened perception of pain. These anxiety-associated symptoms can adversely affect your preparation for surgery; therefore, we recommend reducing tension and anxiety as much as possible.

Unfortunately, there is no miracle cure for overcoming anxiety, but many methods can help you cope with the stress associated with surgery. In addition to being close to family and friends, relaxation exercises are recommended, such as repeating slow and deep inhalations, walking, and seeking outdoor activities. The calming and stress-reducing effect of music has been proven, therefore listening to pleasant, soothing music and practicing meditation are recommended. As your circumstances allow, muscle relaxation exercises, massage, or aromatherapy may also be helpful.

5. ACTIVITY LOG

Week 1

Day / Date	Supplements	Exercises	Additional activity
Day 1<br>2017. __.__	SUPPLEMENTS:<br>________________: □ □ □<br>________________: □ □ □<br>________________: □ □ □	Breathing exercises (10 min): □ 10 points<br>Spirometer (4x10 inhalations): □ □ □ □<br>Value (ml): ______________________<br>Walk (30 min): □ 30 points	___________________________________<br>___________________________________<br>___________________________________
Day 2<br>2017. __.__	SUPPLEMENTS:<br>________________: □ □ □<br>________________: □ □ □<br>________________: □ □ □	Breathing exercises (10 min): □ 10 points<br>Spirometer (4x10 inhalations): □ □ □ □<br>Value (ml): ______________________<br>Walk (30 min): □ 30 points	___________________________________<br>___________________________________<br>___________________________________
Day 3<br>2017. __.__	SUPPLEMENTS:<br>________________: □ □ □<br>________________: □ □ □<br>________________: □ □ □	Breathing exercises (10 min): □ 10 points<br>Spirometer (4x10 inhalations): □ □ □ □<br>Value (ml): ______________________<br>Walk (30 min): □ 30 points	___________________________________<br>___________________________________<br>___________________________________
Day 4<br>2017. __.__	SUPPLEMENTS:<br>________________: □ □ □<br>________________: □ □ □<br>________________: □ □ □	Breathing exercises (10 min): □ 10 points<br>Spirometer (4x10 inhalations): □ □ □ □<br>Value (ml): ______________________<br>Walk (30 min): □ 30 points	___________________________________<br>___________________________________<br>___________________________________
Day 5<br>2017. __.__	SUPPLEMENTS:<br>________________: □ □ □<br>________________: □ □	Breathing exercises (10 min): □ 10 points<br>Spirometer (4x10 inhalations): □ □ □ □	___________________________________<br>___________________________________<br>___________________________________

	□<br>________________: □ □ □	Value (ml):<br>______________________<br>Walk (30 min): □ 30 points	
Day 6<br>2017. __.__	SUPPLEMENTS:<br>________________: □ □ □<br>________________: □ □ □<br>________________: □ □ □	Breathing exercises (10 min): □ 10 points<br>Spirometer (4x10 inhalations): □ □ □ □<br>Value (ml):<br>______________________<br>Walk (30 min): □ 30 points	__________________________________<br>__________________________________<br>__________________________________
Day 7<br>2017. __.__	SUPPLEMENTS:<br>________________: □ □ □<br>________________: □ □ □<br>________________: □ □ □	Breathing exercises (10 min): □ 10 points<br>Spirometer (4x10 inhalations): □ □ □ □<br>Value (ml):<br>______________________<br>Walk (30 min): □ 30 points	__________________________________<br>__________________________________<br>__________________________________

Week 2

Day / Date	Supplements	Exercises	Additional activity
Day 8<br>2017. __.__	SUPPLEMENTS:<br>________________: □ □ □<br>________________: □ □ □<br>________________: □ □ □	Breathing exercises (10 min): □ 10 points<br>Spirometer (4x10 inhalations): □ □ □ □<br>Value (ml):<br>______________________<br>Walk (30 min): □ 30 points	__________________________________<br>__________________________________<br>__________________________________
Day 9<br>2017. __.__	SUPPLEMENTS:<br>________________: □ □ □<br>________________: □ □ □<br>________________: □ □ □	Breathing exercises (10 min): □ 10 points<br>Spirometer (4x10 inhalations): □ □ □ □<br>Value (ml):<br>______________________<br>Walk (30 min): □ 30 points	__________________________________<br>__________________________________<br>__________________________________

Day 10<br>2017. __.__	SUPPLEMENTS:<br>_______________: □ □ □<br>_______________: □ □ □<br>_______________: □ □ □	Breathing exercises (10 min): □ 10 points<br>Spirometer (4x10 inhalations): □ □ □ □<br>Value (ml): ______________________<br>Walk (30 min): □ 30 points	________________________________<br>________________________________<br>________________________________
Day 11<br>2017. __.__	SUPPLEMENTS:<br>_______________: □ □ □<br>_______________: □ □ □<br>_______________: □ □ □	Breathing exercises (10 min): □ 10 points<br>Spirometer (4x10 inhalations): □ □ □ □<br>Value (ml): ______________________<br>Walk (30 min): □ 30 points	________________________________<br>________________________________<br>________________________________
Day 12<br>2017. __.__	SUPPLEMENTS:<br>_______________: □ □ □<br>_______________: □ □ □<br>_______________: □ □ □	Breathing exercises (10 min): □ 10 points<br>Spirometer (4x10 inhalations): □ □ □ □<br>Value (ml): ______________________<br>Walk (30 min): □ 30 points	________________________________<br>________________________________<br>________________________________
Day 13<br>2017. __.__	SUPPLEMENTS:<br>_______________: □ □ □<br>_______________: □ □ □<br>_______________: □ □ □	Breathing exercises (10 min): □ 10 points<br>Spirometer (4x10 inhalations): □ □ □ □<br>Value (ml): ______________________<br>Walk (30 min): □ 30 points	________________________________<br>________________________________<br>________________________________
Day 14<br>2017. __.__	SUPPLEMENTS:<br>_______________: □ □ □<br>_______________: □ □ □<br>_______________: □ □ □	Breathing exercises (10 min): □ 10 points<br>Spirometer (4x10 inhalations): □ □ □ □<br>Value (ml): ______________________<br>Walk (30 min): □ 30 points	________________________________<br>________________________________<br>________________________________

Week 3

Day / Date	Supplements	Exercises	Additional activity
Day 15<br>2017. __.__	SUPPLEMENTS:<br>_______________: □ □ □<br>_______________: □ □ □<br>_______________: □ □ □	Breathing exercises (15 min): □ 15 points<br>Spirometer (5x10 inhalations): □ □ □ □ □<br>Value (ml): ____________________<br>Walk (30 min): □ 30 points	________________________________<br>________________________________<br>________________________________
Day 16<br>2017. __.__	SUPPLEMENTS:<br>_______________: □ □ □<br>_______________: □ □ □<br>_______________: □ □ □	Breathing exercises (15 min): □ 15 points<br>Spirometer (5x10 inhalations): □ □ □ □ □<br>Value (ml): ____________________<br>Walk (30 min): □ 30 points	________________________________<br>________________________________<br>________________________________
Day 17<br>2017. __.__	SUPPLEMENTS:<br>_______________: □ □ □<br>_______________: □ □ □<br>_______________: □ □ □	Breathing exercises (15 min): □ 15 points<br>Spirometer (5x10 inhalations): □ □ □ □ □<br>Value (ml): ____________________<br>Walk (30 min): □ 30 points	________________________________<br>________________________________<br>________________________________
Day 18<br>2017. __.__	SUPPLEMENTS:<br>_______________: □ □ □<br>_______________: □ □ □<br>_______________: □ □ □	Breathing exercises (15 min): □ 15 points<br>Spirometer (5x10 inhalations): □ □ □ □ □<br>Value (ml): ____________________<br>Walk (30 min): □ 30 points	________________________________<br>________________________________<br>________________________________
Day 19<br>2017. __.__	SUPPLEMENTS:<br>_______________: □ □ □<br>_______________: □ □ □<br>_______________: □ □	Breathing exercises (15 min): □ 15 points<br>Spirometer (5x10 inhalations): □ □ □ □ □<br>Value (ml): ____________________	________________________________<br>________________________________<br>________________________________

	□	Walk (30 min): □ 30 points	
Day 20<br>2017. __.__	SUPPLEMENTS:<br>_______________: □ □ □<br>_______________: □ □ □<br>_______________: □ □ □	Breathing exercises (15 min): □ 15 points<br>Spirometer (5x10 inhalations): □ □ □ □ □<br>Value (ml): _____________________<br>Walk (30 min): □ 30 points	________________________________<br>________________________________<br>________________________________
Day 21<br>2017. __.__	SUPPLEMENTS:<br>_______________: □ □ □<br>_______________: □ □ □<br>_______________: □ □ □	Breathing exercises (15 min): □ 15 points<br>Spirometer (5x10 inhalations): □ □ □ □ □<br>Value (ml): _____________________<br>Walk (30 min): □ 30 points	________________________________<br>________________________________<br>________________________________

Week 4

Day / Date	Supplements	Exercises	Additional activity
Day 22<br>2017. __.__	SUPPLEMENTS:<br>_______________: □ □ □<br>_______________: □ □ □<br>_______________: □ □ □	Breathing exercises (15 min): □ 15 points<br>Spirometer (5x10 inhalations): □ □ □ □ □<br>Value (ml): _____________________<br>Walk (30 min): □ 30 points	________________________________<br>________________________________<br>________________________________
Day 23<br>2017. __.__	SUPPLEMENTS:<br>_______________: □ □ □<br>_______________: □ □ □<br>_______________: □ □ □	Breathing exercises (15 min): □ 15 points<br>Spirometer (5x10 inhalations): □ □ □ □ □<br>Value (ml): _____________________<br>Walk (30 min): □ 30 points	________________________________<br>________________________________<br>________________________________
Day 24	SUPPLEMENTS:	Breathing exercises (15	________________________________

2017. __.__	_______________: □ □ □<br>_______________: □ □ □<br>_______________: □ □ □	min): □ 15 points<br>Spirometer (5x10 inhalations): □ □ □ □ □<br>Value (ml):<br>______________________<br>Walk (30 min): □ 30 points	_________________________________<br>_________________________________
Day 25<br>2017. __.__	SUPPLEMENTS:<br>_______________: □ □ □<br>_______________: □ □ □<br>_______________: □ □ □	Breathing exercises (15 min): □ 15 points<br>Spirometer (5x10 inhalations): □ □ □ □ □<br>Value (ml):<br>______________________<br>Walk (30 min): □ 30 points	_________________________________<br>_________________________________<br>_________________________________
Day 26<br>2017. __.__	SUPPLEMENTS:<br>_______________: □ □ □<br>_______________: □ □ □<br>_______________: □ □ □	Breathing exercises (15 min): □ 15 points<br>Spirometer (5x10 inhalations): □ □ □ □ □<br>Value (ml):<br>______________________<br>Walk (30 min): □ 30 points	_________________________________<br>_________________________________<br>_________________________________
Day 27<br>2017. __.__	SUPPLEMENTS:<br>_______________: □ □ □<br>_______________: □ □ □<br>_______________: □ □ □	Breathing exercises (15 min): □ 15 points<br>Spirometer (5x10 inhalations): □ □ □ □ □<br>Value (ml):<br>______________________<br>Walk (30 min): □ 30 points	_________________________________<br>_________________________________<br>_________________________________
Day 28<br>2017. __.__	SUPPLEMENTS:<br>_______________: □ □ □<br>_______________: □ □ □<br>_______________: □ □ □	Breathing exercises (15 min): □ 15 points<br>Spirometer (5x10 inhalations): □ □ □ □ □<br>Value (ml):<br>______________________<br>Walk (30 min): □ 30 points	_________________________________<br>_________________________________<br>_________________________________

FURTHER INFORMATION - CONTACTS

If you have any questions during the treatment or the preoperative preparation program, or if there is any change in your health status, please contact us with confidence using one of the options below.

SZENT BORBÁLA HOSPITAL, TATABÁNYA - DEPARTMENT OF SURGERY AND VASCULAR SURGERY

2800 Tatabánya, Dózsa György út 77.

"H" building, 4th floor, wing 1
Nurses' station: +36 34 515 488 / 377

"H" building, 4th floor, wing 3
Nurses' station: +36 34 515 488 / 384

Outpatient clinic: +36 34 515 488 / 120

Office: +36 34 515 407

STUDY LEAD

Balázs Bánky, MD, PhD +36 20 823 1642
