# Supplementary material for: Identifying Patients with Colorectal Cancer Likely to Benefit from a Trimodal Prehabilitation Prior to Surgery
Source: Nutrients. 2026 Apr 27;18(9):1369. doi: 10.3390/nu18091369 (PMC13164859; doi:10.3390/nu18091369)
Supplement: Supplementary file 1 [file nutrients-18-01369-s001.zip › Prehab-study-supplementary-File S3.pdf]

ERAS												
	Morbidity 7d yes	p value	Morbidity 30d yes	p value	Mortality 7 d yes	p value	Mortality 30 d yes	p value	Morbidity CD3+ 7d yes	p value	Morbidity CD3+ 30d yes	p value
BMI <30	10/69	0,63[a]	13/69	0,33 [a]	0/69	0,10 [b]	0/69	**0,03 [b]**	1/69	0,24 [b]	13/69	0,33 [a]
BMI ≥30	6/33		9/33		2/33		3/33		2/33		9/33	
Male	11/56	0,19 [a]	15/56	0,22 [a]	2/56	0,50 [b]	2/56	0,65 [a]	2/56	1,00 [b]	3/56	1 [b]
Female	5/48		8/48		0/48		1/48		1/48		2/48	
Age <65 years	7/31	0,18[a]	9/31	0,28 [a]	1/31	0,51 [b]	1/31	0,89 [a]	0/31	0,55 [b]	1/31	1 [b]
Age ≥65 years	9/73		14/73		1/73		2/73		3/73		4/73	
CR-POSSUM <3	13/79	0,76 [b]	18/79	0,84 [a]	2/79	1 [b]	2/79	0,55 [b]	2/79	0,55 [b]	3/79	0,33 [b]
CR-POSSUM ≥3	3/24		5/24		0/24		1/24		1/24		2/24	
6 MWD <160 m	5/23	0,30 [a]	5/23	0,92 [a]	1/23	0,41 [b]	1/23	0,41 [b]	1/23	0,54 [b]	1/23	1 [a]
6 MWD ≥160 m	10/77		16/77		1/77		1/77		1/77		3/74	
FVC <3100 ml	3/29	0,29 [a]	4/29	0,19 [a]	0/29	na	0/29	na	0/29	1	0/29	1 [a]
FVC ≥3100 ml	8/41		11/41		0/41		0/41		1/41		1/41	
Colon lesion	4/43	0,26 [b]	6/43	0,11 [a]	0/43	0,51 [b]	0/43	0,26 [b]	1/43	1	1/43	0,39 [b]
Rectum lesion	11/59		16/59		2/59		3/59		2/59		4/59	
Non-myosteatotic	3/42	**0,03 [a]**	4/42	**0,01 [a]**	0/42	0,14 [b]	0/42	0,52 [b]	0/42	0,14 [b]	0/42	0,52 [b]
Myosteatotic	7/26		11/26		2/26		24/42		2/26		3/26	
Frailty low	7/27	0,13 [a]	9/27	0,18 [a]	1/27	0,59 [a]	1/27	0,91 [a]	1/27	0,91 [a]	1/27	0,85 [a]
Frailty medium	4/47		7/47		1/47		1/47		1/47		2/47	
Frailty high	5/30		7/30		0/30		1/30		1/30		2/30	
Sarcopenia no	6/28	0,48 [b]	9/28	0,34 [b]	1/28	1 [b]	2/28	0,49 [b]	1/28	1 [b]	2/28	1 [b]
Sarcopenia yes	3/23		4/23		0/23		0/23		1/23		1/23	
MUST score low	0/12	0,27 [a]	0/12	0,13 [a]	0/12	0,23 [a]	0/12	0,60 [a]	0/12	0,72 [a]	0/12	0,49 [a]
MUST score medium	7/41		9/41		2/41		2/41		1/41		1/41	
MUST score high	9/48		13/48		0/48		1/48		2/48		3/48	

[a]: khi2 test; [b]: Fisher exact test

Subgroup analysis within the ERAS subcohort, grouped according to study outcomes.

Prehabilitation

	Morbidity 7d yes	p value	Morbidity 30d yes	p value	Mortality 7 d yes	p value	Mortality 30 d yes	p value	Morbidity CD3+ 7d yes	p value	Morbidity CD3+ 30d yes	p value
BMI <30	7/87	**0,03** [a]	10/87	**0,01** [a]	0/87	na	0/87	0,36 [b]	1/87	0,05 [b]	2/87	0,09 [b]
BMI ≥30	10/48		18/48		0/48		1/48		4/48		5/48	
Male	7/72	0,37 [a]	12/72	0,11 [a]	0/72	na	1/72	1 [b]	2/72	0,67 [b]	2/72	0,09 [b]
Female	10/68		19/68		0/68		0/68		3/68		7/68	
Age <65 years	7/44	0,36 [a]	10/44	0,91 [a]	0/44	na	1/44	0,31 [b]	1/44	1 [b]	1/44	0,27 [b]
Age ≥65 years	10/96		21/96		0/96		0/96		4/96		8/96	
CR-POSSUM <3	13/104	1 [b]	25/104	0,36 [a]	0/104	na	1/104	1 [b]	4/104	1 [b]	8/104	0,45 [b]
CR-POSSUM ≥3	4/36		6/36		0/36		0/36		1/36		1/36	
6 MWD <160 m	4/37	1 [b]	10/37	0,42 [a]	0/37	na	0/37	1 [b]	1/37	1 [b]	3/37	0,70 [b]
6 MWD ≥160 m	13/102		21/102		0/102		1/102		4/102		6/102	
FVC <3100 ml	7/38	0,75 [b]	9/38	0,89 [a]	0/38	na	0/38	0,48 [b]	2/38	1 [b]	3/38	0,62 [b]
FVC ≥3100 ml	5/36		9/36		0/36		1/36		1/36		1/36	
Colon lesion	6/67	0,24 [a]	14/67	0,81 [a]	0/67	na	0/67	1 [b]	1/67	0,37 [b]	3/67	0,72 [b]
Rectum lesion	11/71		16/71		0/71		1/71		4/71		5/71	
Non-myosteatotic	9/46	0,16 [a]	12/46	0,59 [a]	0/46	na	0/46	na	2/46	1 [b]	3/46	0,73 [b]
Myosteatotic	6/60		13/60		0/60		0/60		3/60		6/60	
Frailty low	7/45	0,16 [a]	8/45	0,17 [a]	0/45	na	0/45	0,10 [a]	1/45	**0,01** [b]	3/45	**0,01** [b]
Frailty medium	5/70		14/70		0/70		0/70		0/70		1/70	
Frailty high	5/25		9/25		0/25		1/25		4/25		5/25	
Sarcopenia no	5/25	0,79 [a]	8/25	0,52 [a]	0/25	na	0/25	na	2/25	0,59 [b]	2/25	1 [b]
Sarcopenia yes	5/29		7/29		0/29		0/29		1/29		2/29	
MUST score low	3/24	0,61 [a]	5/24	0,56 [a]	0/24	na	0/24	0,55 [a]	1/24	0,47 [a]	2/24	0,83 [a]
MUST score medium	6/62		11/62		0/62		1/62		1/62		3/62	
MUST score high	8/50		13/50		0/50		0/50		3/50		3/50	

[a]: khi2 test; [b]: Fisher exact test

Subgroup analysis within the prehabilitation subcohort, grouped according to study outcomes.
